# Drug Safety in Hospitalized Diabetes Patients: A Retrospective Analysis of Predictors and Clinical Relevance of Potential Drug–Drug Interactions

**DOI:** 10.3390/healthcare14091224

**Published:** 2026-05-02

**Authors:** Muhammad Adil Khan, Nadia Farhanah Syafhan, Sidra Noor, Mohammed S. Alshammari, Meshal Alotaibi, Waad Alrohily, Abdulaziz H. Alanazi, Wael A. Alsubhi, Latifah Al Shammari, Mohd Rasheeduddin Imran, Ashfaq Ahmad

**Affiliations:** 1 Department of Clinical Pharmacy and Pharmacy Practice Research Cluster, Faculty of Pharmacy, Universitas Indonesia, Depok 16424, Indonesia; adilmrd0344@gmail.com (M.A.K.); nadia.farhanah@farmasi.ui.ac.id (N.F.S.); 2 Department of Pharmacy Practice, Shifa College of Pharmaceutical Sciences, Shifa Tameer-e-Millat University, Islamabad 44000, Pakistan; sidranoordop@gmail.com; 3 Department of Pharmacy Practice, College of Pharmacy, University of Hafr Al Batin, Hafr Al Batin 39524, Saudi Arabia; moalshammari@uhb.edu.sa (M.S.A.); motaibi@uhb.edu.sa (M.A.); waalsubhi@uhb.edu.sa (W.A.A.); dr.imran@uhb.edu.sa (M.R.I.); 4 Department of Pharmacy Practice, College of Pharmacy, Taibah University, Medina 52571, Saudi Arabia; wrohily@taibahu.edu.sa; 5 Department of Clinical Practice, College of Pharmacy, Northern Border University, Rafha 76313, Saudi Arabia; abdulaziz.alanazi4@nbu.edu.sa; 6 Department of Pharmaceutical Chemistry, College of Pharmacy, University of Hafr Al Batin, Hafr Al Batin 39524, Saudi Arabia; lamalshammari@uhb.edu.sa

**Keywords:** type 2 diabetes mellitus (T2DM), potential drug–drug interaction, clinical relevance, patient safety

## Abstract

**Background**: Diabetes mellitus is frequently associated with complications and comorbidities that often require hospitalization and the use of multiple medications for effective management. However, the simultaneous use of these treatments significantly increases the risk of potential drug–drug interactions (pDDIs). **Objectives**: This study assessed the prevalence, levels, and associated predictors of pDDIs among hospitalized participants with type 2 diabetes mellitus (T2DM) and evaluated their clinical relevance and implications for monitoring and management. **Methods**: This retrospective cross-sectional study included 430 inpatients with T2DM at Universitas Indonesia Hospital, Indonesia. Lexicomp^®^ Lexi-Interact™ software Wolters Kluwer was used to analyze and classify pDDIs based on severity, risk rating, and documentation levels. Additionally, logistic regression analysis was conducted to identify the predictors of pDDIs, and the study assessed the clinical relevance of major pDDIs. **Results**: Of the total participants, 84.7% (*n* = 364) experienced pDDIs, with 1642 interactions identified. Moderate interactions accounted for 77.5% (*n* = 1273), whereas major interactions constituted 12.2% (*n* = 201). The most common risk rating was category C (77.5%, *n* = 1187), and the predominant evidence support level was ‘fair’ (64.8%, *n* = 1064). Multivariate logistic regression analysis showed a significant association between pDDIs and of 7–12 medications used (OR = 30.1; *p* < 0.001), and hospital stays ≥4 days (OR = 9.7; *p* = 0.001). Major pDDIs were significantly linked to ≥13 medications (OR = 5.5; *p* = 0.002), ≥4 days hospitalization (OR = 11.3; *p* < 0.001), and urinary tract infections (OR = 3.5; *p* = 0.02). Participants with major pDDIs exhibited hypoglycemia, hyperglycemia, electrolyte imbalances, and reduced therapeutic responses. **Conclusions**: The findings indicate a high prevalence of pDDIs among participants with T2DM, highlighting the impact of polypharmacy, prolonged hospitalization, and comorbidities. Implementing software-based screening, close monitoring, and targeted interventions are essential to reduce adverse clinical outcomes and enhance patient safety.

## 1. Introduction

Diabetes mellitus is a chronic metabolic disorder that is rapidly transforming into a global health crisis, with its alarming prevalence threatening millions of lives and straining healthcare systems worldwide [1]. The International Diabetes Federation (IDF) reported that approximately 536.6 million individuals are living with diabetes worldwide, which is projected to increase to 783 million by 2045 [2]. In Indonesia, Basic Health Research statistics reveal a significant increase in diabetes cases, from 18.69 million in 2020 to 23.2 million by 2030 and 40.7 million by 2045. Although these estimates encompass all forms of diabetes, they predominantly reflect type 2 diabetes mellitus (T2DM), which accounts for approximately 90–95% of cases globally and regionally. This upward trend in the prevalence of diabetes highlights the public health challenge of T2DM in Indonesia [3].

Patients with diabetes frequently experience multiple complications, including microvascular disorders, such as diabetic retinopathy, nephropathy, and neuropathy, as well as macrovascular complications, such as coronary artery disease, cerebrovascular disease, and peripheral artery disease [4]. Additionally, they often experience comorbidities, such as hypertension, obesity, chronic kidney disease (CKD), gastrointestinal disorders, and urinary tract infections (UTIs), further complicating disease management [5]. Effective management of diabetes and its associated complications and comorbidities is crucial to slow disease progression and reduce mortality [6]. To address these complexities, single-drug therapies are generally inadequate, necessitating combination treatments to achieve optimal glycemic control while effectively managing comorbidities and complications, which further complicates pharmacological treatment [7]. However, these combination treatments often increase the risk of drug–drug interactions, adding another layer of complexity to the pharmacological management of diabetes and its associated conditions [8].

Drug–drug interactions (DDIs) refer to combinations of drugs that can alter the pharmacokinetic parameters or pharmacodynamic profiles of one or both drugs [9]. These interactions may result in negative clinical consequences, including reduced or diminished therapeutic efficacy, toxicity, adverse effects, and hospitalization [10]. DDIs are responsible for 20–30% of reported adverse effects, with 1–2% being life-threatening and 70% requiring clinical intervention [11]. However, DDIs are often both predictable and preventable [12]. Older age, comorbidities, longer hospital stays, and a higher number of prescribed medications are key predictors of DDIs [13]. In patients with diabetes, the risk of DDIs and their associated adverse outcomes is higher because they frequently use multiple medications concurrently [14]. Moreover, therapeutic options for managing diabetes mellitus have significantly expanded in recent decades, with increasing diversity in mechanisms of action and growing availability due to favorable therapeutic outcomes and cost-effectiveness [15].

DDIs was not only studied among patients with diabetes [16,17] but their knowledge and awareness gaps among medical and non-medical students were also studied [18]. However, research in this area remains limited in certain regions, and existing studies are often constrained by specific scopes and limitations, such as involving small sample sizes and using different drug interaction checker tools [19]. Some studies have focused exclusively on the prevalence of DDIs [20], whereas others have examined factors related to their occurrence [1]. Additionally, a subset of research has been restricted to evaluating DDI specifically between antidiabetic agents, neglecting potential interactions with concomitant medications [21,22,23]. Most studies are further confined to outpatient settings, emphasizing medication safety [16,24].

DDIs involving antidiabetic agents and concomitant medications used for managing comorbidities and complications pose a major concern in clinical practice because of their potential to cause severe adverse effects [16]. Moreover, knowledge of the most common major interacting pairs used in patients with diabetes, along with identifying pDDI predictors, is essential to reducing drug-related problems and improving therapeutic compliance and outcomes [25]. A high prevalence of pDDIs was observed in this study, with polypharmacy and prolonged hospitalization as major contributing factors. In addition, this study will contribute to the advancement of rational drug use and the prevention and management of drug interactions, leading to enhanced therapeutic outcomes and improved quality of life for patients.

Therefore, this study aimed to assess the prevalence and levels of pDDIs, and identify the predominant factors associated with their occurrence among diabetic inpatients in a tertiary care setting. Additionally, this study sought to evaluate the clinical relevance of the most frequent major pDDIs, and their implications for monitoring and management. A better understanding of the predictors and clinical relevance of major interactions could help prescribe strategies and policies to prevent or mitigate the adverse outcomes of pDDIs and enhance patient safety and therapeutic outcomes.

## 2. Materials and Methods

### 2.1. Study Design and Setting

This analytical cross-sectional study was conducted retrospectively at the Universitas Indonesia Hospital (RSUI), one of the region’s largest university-affiliated hospitals located just south of Jakarta, the capital of Indonesia. RSUI is a class A general hospital with over 300 beds, offering specialized and subspecialty medical services. As a referral center, it accepts patients from various healthcare facilities. As a university-affiliated teaching hospital, it is more convenient to collect data. Furthermore, there is no specific criteria for DDI screening in the hospital setting.

The ethical review board of Rumah Sakit Universitas Indonesia approved this study with reference No. (S-025/RSUI, dated 10 February 2024). As this research solely used patients’ medical records, obtaining individual informed consent was waived by the ethical review board.

### 2.2. Study Participants

The study included participants admitted to the inpatient department of RSUI with type 2 diabetes mellitus. Medical records from January 2022 to December 2023 were retrospectively reviewed to identify eligible participants. Participants were included if they were 18 years or older and prescribed two or more medications concurrently during hospitalization. Participants with type 1 diabetes, those who were admitted to the intensive care unit (ICU) due to more complex treatment protocols for recovery [26].

**Figure 1:**
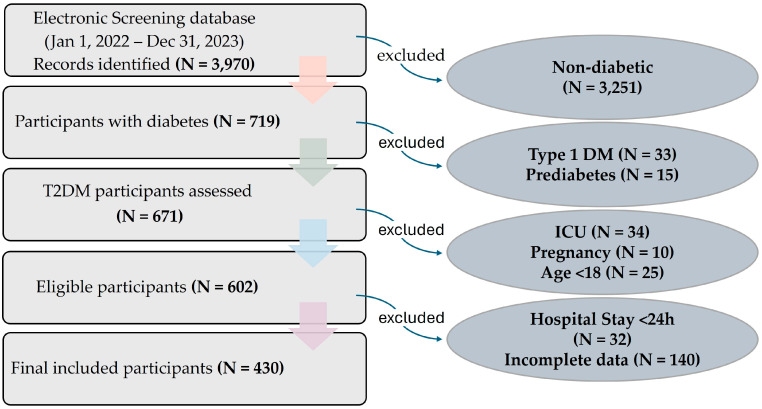
Flow chart of study cohort selection.

Pregnant participants and those with incomplete medical records (e.g., missing gender, age, diagnosis, laboratory results, or prescribed medications) were excluded from the study. The screening and selection process is summarized in Figure 1, illustrating the number of participants screened, excluded, and included in the final analysis.

### 2.3. Data Source and Collection

Initially, electronic medical records were screened to identify all participants who met the inclusion and exclusion criteria (Figure 1), ensuring a total sampling approach. This approach minimized selection bias and provided comprehensive coverage of the target population. The profile of participants who met the inclusion criteria were systematically reviewed, and detailed information was collected from their medical records. This included demographic data (age, gender), diagnosis, chief complaints, laboratory investigations, clinical signs/symptoms, details of prescribed medications (dose, duration, dosage form, and route of administration), and the total number of prescribed medicines during their hospital stay, recorded using daily progress sheets. All data were organized and recorded in a structured Excel format for further analysis.

### 2.4. Medications Profiles Screening for pDDIs

To screen the prescribed medications for pDDIs, the lexicomp^®^UptToDate drug interaction checker was used. This subscription-based tool systematically classifies drug interactions based on severity level, including major (high clinical risk; may be life-threatening or cause serious harm), moderate (may worsen clinical condition and require treatment adjustment), and minor (minimal clinical impact with limited effects); risk rating categories: X (avoid combination), D (consider therapy modification), C (monitor therapy), and B (no action required), and documentation levels (excellent: interaction is well established by controlled studies; good: strong supporting evidence exists but controlled trials are lacking; fair: limited evidence). It also provides reliable scientific evidence and comprehensive information about potentially interacting drugs based on the published literature. Several studies have evaluated the performance of Lexi-interact and recognized it as a high-drug screening software and reported that Lexi-Interact is a reliable drug interaction screening tool, demonstrating high specificity (80–90%) and sensitivity (87–90%) software [27,28]. The overall prevalence of potential drug–drug interactions (pDDIs), as well as their prevalence categorized by severity level, risk rating, and documentation level, was reported. These findings were derived from an analysis of drug pairs identified in each prescription.

### 2.5. Clinical Relevance and Causality Assessment

The clinical relevance of the top 10 major pDDIs was assessed using guidelines developed through a comprehensive review of relevant literature [29]. This analysis was limited to the most frequent major pDDIs, as these interactions are more likely to result in clinically significant outcomes and have a greater impact on patient safety. Each participant’s profile was evaluated for potential adverse outcomes, including abnormal signs and symptoms and expected laboratory abnormalities that may indicate adverse effects. The adverse drug events in this study were classified based on the following specified criteria.

- Leukocytosis an abnormal increase in leukocytes in the blood > 11,000/μL;
- Elevated blood urea nitrogen (BUN) level: BUN > 20 mg/dL
- Elevated serum creatinine levels: serum creatinine > 1.00 mg/dL
- Increase d-Dimer level: >500 ng/mL
- Hyperkalemia: serum potassium level exceeding 5.5 mmol/L.
- Hypokalemia: serum potassium level below 3.5 mmol/L
- Hyponatremia: serum sodium level below 135 mmol/L
- Eosinopenia: lower-than-normal level of eosinophils in the blood < 40 cells/µL
- Neutrophilic leukocytosis: increase in leukocytes count > 11,000/μL as well as absolute neutrophil count (ANC) increases >70%.
- Thrombocytopenia: decreased platelet counts below 150,000/µL.
- Prolonged prothrombin time (PT), >15.5 s.
- Hypoglycemia: fasting blood sugar level (FBS) < 60 mg/dL and random blood sugar level (RBS) < 70 mg/Dl.
- Bradycardia: heart rate less than 60 beats/min.
- Tachycardia: heart rate greater than 100 beats/min;
- Hypotension: systolic blood pressure < 80 mm Hg and/or a diastolic blood pressure < 50 mm Hg.

The drug interaction probability scale (DIPS) was used for the top 10 major pDDIs to evaluate the likelihood of adverse outcomes resulting from pDDIs. This tool systematically identifies and categorizes the causality of adverse effects in patients exposed to interacting medications. Based on the DIPS scoring system, outcomes were classified into four categories: highly probable (score > 8), probable (score 5–8), possible (score 2–4), and doubtful (score < 2) [30].

### 2.6. Variables

Demographic and clinical variables were obtained from medical records. The demographic variables included gender and age. Age was categorized into 18–60 years and >60 years. Clinical variables included the number of medications used, length of hospital stay, number of comorbidities, and most prevalent comorbidities or complications observed. These comorbidities included hypertension, diabetes complications, chronic kidney disease (CKD), congestive heart failure (CHF), coronary artery disease (CAD), acute kidney injury (AKI), cerebrovascular disease (CSVD), dyslipidemia, and urinary tract infections (UTIs). The dependent variables were exposure to pDDIs of any severity (major, moderate, and minor) and exposure to major pDDIs. The independent variables included gender, age, number of medications used, length of hospital stay, number of comorbidities, and identified prevalent comorbidities.

### 2.7. Statistical Analysis

Initially, we analyzed the descriptive statistics for all participants, and the data were presented as frequencies and percentages. The participants were divided into two groups based on their exposure to pDDIs: those exposed to all types of pDDIs and those exposed to major pDDIs. The demographic and clinical characteristics of the participants were compared between the two groups. Cross-tabulations and Pearson’s chi-square test or Fisher’s exact test were applied, as appropriate, to assess the significance of differences between groups. Binary logistic regression analysis explored the relationship between pDDI exposure and independent variables. Univariate analysis was conducted first, followed by multivariate analysis for variables with univariate *p*-values ≤ 0.15. Odds ratios (OR) and 95% confidence intervals (CI) were calculated to quantify these relationships. A *p*-value of ≤0.05 was considered statistically significant. All analyses were performed using SPSS version 28.

## 3. Results

### 3.1. Demographic and Clinical Characteristics of Hospitalized Participants with Diabetes and Their Exposure to Any Type pDDIs and Major pDDIs

Table 1 shows the characteristics of the participants. A total of 430 participants with diabetes were included in the data analysis, of which 232 (54%) were female and 198 (46%) were male. Most (54.2%) participants were aged > 60 years. The majority (54.4%) of participants were prescribed 7–12 medications. Most participants (54.7%) stayed in the hospital for 3 or less days. More than half of the participants (70.9%) had two or fewer comorbidities.

Hypertension (*n* = 132), diabetes complications (71), and chronic kidney disease (CKD) (48) were the three prevalent comorbidities in the study population. All participants demographic and clinical characteristics are shown in Table 1, with separate data and analysis for patients exposed to pDDIs of all types and major pDDIs. There is statistically significant association of all types of pDDIs with the number of medicines per patient (*p* < 0.001), hospital stay (<0.001), number of comorbidities (<0.001), hypertension (0.001), and AKI (0.03) (Table 1). Furthermore, statistically significant relationship was observed for major-PDDIs with age (*p* = 0.05), number of medicines (<0.001), hospital stay (<0.001), number of comorbid illnesses (< 0.001), hypertension (<0.001), CHF (0.04), and CSVD (0.005) (Table 1).

### 3.2. Prevalence and Classification Levels of pDDIs

**Figure 2:**
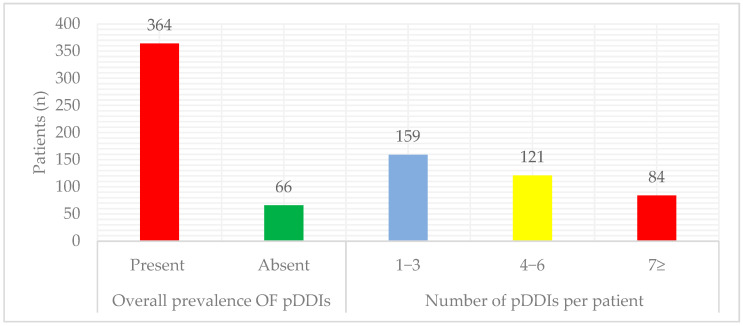
Prevalence of potential drug–drug interactions in participants with T2DM. Data are presented as frequencies. Overall prevalence is the occurrence of at least one pDDIs. The total number of participants was 430; therefore, the overall prevalence of pDDIs was 84.7% (364/430).

**Figure 3:**
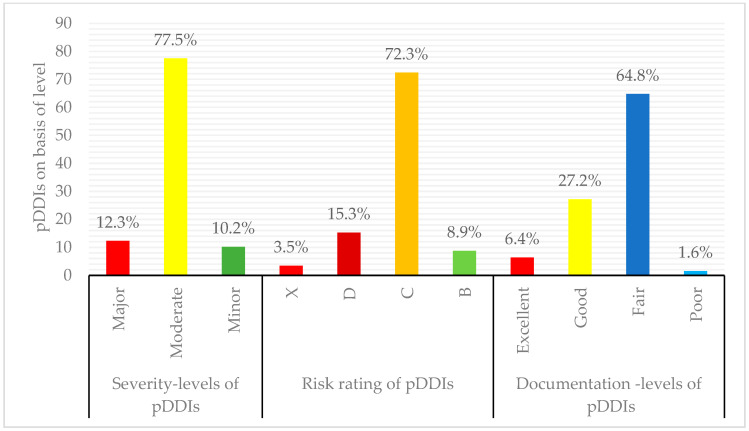
Distribution of identified pDDIs in participants with T2DM according to severity, risk rating, and documentation levels (*n* = 1642). Data are presented as percentages.

Among 430 diabetic participants (Figure 2), pDDIs were identified in 364 participants (84.7%), with 159 (52.3%) experiencing 1–3 interactions, 121 (28.2%) having 4–6 interactions, and 84 (19.5%) reporting ≥7 interactions. A total of 1642 pDDIs were identified, with 77.5% classified as moderate severity, 12.2% as major, and 10.2% as minor. Regarding risk ratings, 77.5% were in Category C, 15.3% in Category D, 8.9% in Category B, and 3.5% in Category X. In terms of documentation, 64.8% had fair scientific evidence, 27.2% had good evidence, 6.4% had excellent evidence, and 1.6% were poor (Figure 3).

### 3.3. Predictors of pDDIs in Participants with T2DM

The predictors of pDDIs in participants with T2DM are presented in Table 2. The results of bivariate logistic regression analysis identified significant associations between the presence of pDDIs and the use of 7–12 medications (OR = 44.5; 95% CI = 18.1–109.3; *p* < 0.001) and ≥13 medications (OR = 20.8; 95% CI = 7.8–55.6; *p* < 0.001), hospital stays of ≥4 days (OR = 23.4; 95% CI = 7.2–76; *p* < 0.001), presence of ≥3 comorbidities (OR = 6; 95% CI = 2.3–15.3; *p* < 0.001), hypertension (OR = 3.2; 95% CI = 1.5–6.7; *p* = 0.002), acute kidney injury (AKI) (OR = 6.7; 95% CI = 0.9–49.8; *p* = 0.06), and gastroenteritis (OR = 0.5; 95% CI = 0.2–1.3; *p* = 0.14). In the multivariate logistic regression analysis, the association remained significant with the use of 7–12 medications (OR = 30.1; 95% CI = 11.7–77.8; *p* < 0.001), and hospital stays of ≥4 days (OR = 9.7; 95% CI = 2–38.7; *p* = 0.001).

Similarly, the predictors of major pDDIs are presented in Table 2. Univariate logistic regression analysis identified significant associations between major pDDIs and age ≥ 60 years (OR = 1.5; 95% CI = 0.9–2.2; *p* = 0.05), the use of 7–12 medications (OR = 6.8; 95% CI = 2.9–16.3; *p* < 0.001) and ≥13 medications (OR = 28.2; 95% CI = 11.2–71.2; *p* < 0.001), hospital stays of ≥4 days (OR = 18.5; 95% CI = 10.6–32.3; *p* < 0.001), and the presence of ≥3 comorbidities (OR = 4.6; 95% CI = 2.9–7.2; *p* < 0.001). Significant associations were also observed for hypertension (OR = 2.1; 95% CI = 1.3–3.2; *p* < 0.001), congestive heart failure (CHF) (OR = 1.9; 95% CI = 1.0–3.9; *p* = 0.04), cerebral small vessel disease (CSVD) (OR = 2.9; 95% CI = 1.6–6.2; *p* = 0.007), and urinary tract infection (UTI) (OR = 2; 95% CI = 0.9–4.7; *p* = 0.07). Multivariate logistic regression analysis confirmed significant associations for major pDDIs with prescriptions of ≥13 medications (OR = 5.5; 95% CI = 1.9–16.1; *p* = 0.002), hospital stays of ≥4 days (OR = 11.3; 95% CI = 5.9–21.6; *p* < 0.001), and UTI (OR = 3.5; 95% CI = 1.1–10.6; *p* = 0.02).

### 3.4. Clinical Relevance and Causality Assessment of the Top 10 Major pDDIs

Table 3 shows the top 10 major drug interactions and their, clinical relevance with relevant signs, symptoms, laboratory reports, and monitoring/management guidelines [29,31]. Participants exposed to ceftriaxone–calcium-containing products exhibited respiratory distress, fever, and signs of sepsis, with laboratory findings of leukocytosis, elevated BUN, and increased serum creatinine. Clopidogrel–omeprazole interactions were associated with cardiovascular symptoms, including shortness of breath, pedal edema, and elevated D-dimer levels. Electrolyte imbalances and renal dysfunction were prevalent in participants with ramipril–spironolactone and spironolactone–candesartan interactions, presenting with hyperkalemia, hyponatremia, elevated BUN, and serum creatinine, accompanied by tachycardia, chest pain, and gastrointestinal symptoms. Similarly, clopidogrel–lansoprazole interactions were linked to cardiovascular complications and coagulation abnormalities. Participants with levofloxacin–sucralfate interactions exhibited infectious complications, including fever and sepsis, whereas aspirin–ketorolac interactions were associated with increased bleeding risk, thrombocytopenia, and prolonged clotting times. Insulin–aspart–pioglitazone interactions lead to hypoglycemic episodes with nervous system symptoms, whereas spironolactone–ketorolac interactions result in hypotension, bradycardia, and renal impairment. Similarly, participants with the allopurinol–ramipril interaction exhibited eosinopenia, leukocytosis, and clinical manifestations such as fever, bradycardia, and acral edema.

According to the drug interaction probability scale (DIPS), a score of 5 was found in the following DDIs as shown in Table 3: ceftriaxone + calcium-containing products (*n* = 4), ramipril + spironolactone (*n* = 2), spironolactone + candesartan (*n* = 2), aspirin + ketorolac (*n* = 1), spironolactone + ketorolac (*n* = 1), insulin–aspart + pioglitazone (*n* = 1), while the, following frequencies of interactions had a score of 4: ceftriaxone + calcium-containing products (*n* = 5), clopidogrel + omeprazole (*n* = 1), ramipril + spironolactone (*n* = 2), spironolactone + candesartan (*n* = 1), levofloxacin + sucralfate (*n* = 1), and aspirin + ketorolac (*n* = 1).

## 4. Discussion

Diabetes mellitus is a leading noncommunicable disease in the modern era, with its prevalence increasing dramatically across the globe [33]. Drug–drug interactions remain an important therapeutic challenge among hospitalized patients with diabetes [34]. This is the first study to focus on the prevalence, categorization, risk factors, and clinical relevance of pDDIs among hospitalized participants with T2DM in Indonesia. Our current study’s prevalence (84.7%) is consistent with findings reported by a retrospective study in China among hospitalized patients with T2DM, which reported a substantial burden of pDDIs and highlighted polypharmacy as a key contributing factor [17]. Similarly, a cross-sectional study from Bangladesh demonstrated that 55.6% of diabetic patients experienced potential drug–drug interactions [35]. Furthermore, comparable high prevalence rates of pDDIs have also been reported in other clinical populations.

Extensive cross-sectional studies from Serbia (among myasthenia gravis), Slovenia (among schizophrenia), and Spain (among chronic CKD patients) have reported prevalence rates ranging from 80 to 91.7% [32,36,37]. While data from a large observational cross-sectional study conducted across 12 Spanish regions, involving 12 hospitals and 21 NGOs from 36 different institutions, among HIV patients indicated that 51.1% of patients were exposed to at least one DDI [38]. Another study conducted in Belgrade, Serbia, among hypertensive patients attending the outpatient department, indicated that 80.6% of patients were exposed to at least one pDDI [39]. However, results from Asian countries have shown variability. A recent retrospective analysis of data from elderly patients (aged ≥ 65 years) in Sri Lanka reported a 77.1% prevalence of pDDIs [40]. Another study from Pakistan reported a 78% prevalence of pDDIs among cancer patients and a 98.7% prevalence among cardiac patients [41,42]. The discrepancies in pDDI prevalence across various studies may be attributed to differences in the study population, study design, drug prescription pattern, and drug interaction screening software. Our findings indicate a higher overall prevalence of pDDIs. Considering the findings of this study, patients with T2DM are at higher risk of DDIs. Moreover, the Indonesian population is at a higher risk of pDDIs, primarily due to the irrational use of medications, unavailability of clinical pharmacy services, and absence of effective DDI screening systems in hospitals [43].

To effectively manage adverse events and reduce/prevent the risks associated with pDDIs, healthcare professionals must accurately identify pDDI levels. In this study, moderate severity (77.5%) pDDIs were the most frequently identified, followed by major (12.3%) severity interactions. Similarly, pDDIs with type C (72.3%) risk ratings were more frequent. The fair (64.8%) documenting type was more prevalent, followed by the good (27.2%) documenting level. These findings are consistent with those of other studies on hospitalized patients with different conditions and specialties. A Pakistani study Among hospitalized patients with chronic kidney disease, patients reported moderate pDDIs (79.0%) and fair (72.0%) documentation-level pDDIs were more frequently observed [44]. Another retrospective cross-sectional study conducted in three hospitals in Bangladesh reported a 63.4% prevalence of moderate pDDIs, whereas 52.7% were categorized as having a fair documentation level [45]. Despite extensive research on pDDIs, reporting risk rating classifications are still limited. The lack of standardized reporting frameworks makes cross-study comparisons difficult, emphasizing the need for further investigation. Additionally, these findings highlight that patients with T2DM are at risk of adverse consequences related to pDDIs. Therefore, properly identifying the pDDI type is important for healthcare professionals because it plays a critical role in the clinical management of pDDIs. Accurate classification is also crucial for designing effective prophylactic measures to prevent and mitigate risks.

Polypharmacy is a growing concern among hospitalized patients with diabetes who frequently receive several medications to treat comorbidities and complications [21]. We also observed a strong association between polypharmacy and the presence of pDDIs, a finding well-supported by previously published studies [41,46]. A study conducted in Pakistan reported that patients taking seven or more medications had a significantly elevated risk of pDDIs, with an odds ratio of 27.63 [47]. Our findings also indicate a significant relationship between pDDIs, and prolonged hospital stays, consistent with prior studies [14,48]. Our results demonstrated that patients receiving 7–12 or ≥13 medications and those hospitalized for ≥4 days were significantly more likely to experience pDDIs, underscoring the combined impact of polypharmacy and prolonged hospitalization on interaction risk. Additionally, the odds of exposure to major pDDIs were separately calculated to provide a more detailed risk assessment. The significant association between major pDDIs and polypharmacy and prolonged duration of hospital stay observed in this study is consistent with the findings of previous research [47,49]. Likewise, another study reported that clinically significant DDI risk increases with the use of five or more medications and prolongs hospitalization [50]. Furthermore, hospitalized patients with T2DM, particularly those at risk of UTIs, frequently require antibiotics, which are a major contributor to clinically relevant DDIs. Healthcare professionals must be aware of the risk factors for pDDIs to ensure careful evaluation and individualization of therapy for patients with diabetes. These tailored treatment strategies can help optimize treatment outcomes while minimizing the risk of developing pDDIs.

This study uniquely linked the potential adverse effects of DDIs with clinical symptoms and abnormal laboratory findings. This approach has rarely been explored in existing literature. Although some studies have examined adverse drug events (ADEs) resulting from DDIs, they often lack detailed monitoring strategies and fail to specify clinically significant interacting drug pairs [51,52,53]. These findings represent a novel approach from a practical perspective and can reduce the risk associated with pDDIs. These considerations provide a better understanding of the negative consequences of interactions. Additionally, monitoring parameters and management strategies will help healthcare professionals accurately assess and effectively manage drug interactions in patients with T2DM.

## 5. Limitations

This study has some limitations. First, this study was conducted at a single center, which, while providing valuable insights, may restrict the generalizability of the findings. A large multicenter study is needed to understand the broader perspective and enhance the applicability of the results to diverse patient populations. Second, the essence of this study was the drug interaction checker. Although a single software was used to screen medication profiles for pDDIs, it is important to acknowledge that variations exist among the available screening tools [54]. Another limitation of this study is that clinical relevance and DIP scale assessment were applied retrospectively, which, while offering valuable insights, could be further enriched by complementing it with prospective studies. Such studies would allow for real-time monitoring and validation of the findings, enhancing the understanding of drug interactions and their clinical impact.

## 6. Conclusions

This study concluded that pDDIs are highly prevalent among hospitalized participants with type 2 diabetes mellitus (84.7%), highlighting their clinical significance and the need for effective management strategies to minimize associated risks. The majority of identified pDDIs were of moderate severity (77.5%), while major interactions accounted for 12.2%, indicating a considerable burden of clinically significant interactions. Polypharmacy, prolonged hospital stays, and multiple comorbidities were identified as key predictors of pDDIs. Multivariate analysis further demonstrated that participants receiving 7–12 medications (OR = 30.1) and ≥13 medications (OR = 5.5) had a significantly increased risk of pDDIs, while hospital stays ≥4 days were strongly associated with both overall pDDIs (OR = 9.7) and major pDDIs (OR = 11.3). Additionally, urinary tract infections were identified as an independent predictor of major pDDIs (OR = 3.5). Moreover, the study linked pDDIs with specific clinical symptoms and abnormal laboratory findings, providing a practical approach to understanding their clinical consequences. Commonly observed adverse effects included hypoglycemia, hyperkalemia, changes in ECG, hepatotoxicity, and subtherapeutic effects. The findings reinforce the need for systematic screening, close monitoring, and individualized medication management to minimize pDDI-related risks. These findings are based on a single-center study and should be interpreted within the context of this hospital setting. Further research is required to assess the clinical, economic, and patient-centered impacts of pDDIs and their burden on Indonesia’s healthcare system. These studies could help develop medication policies, improve prescribing practices, and enhance the management of diabetes care. Ultimately, this research will enhance patient safety and drug therapy, equipping healthcare professionals to manage DDIs better and reduce adverse outcomes.

## Figures and Tables

**Table 1:** The characteristics of the participants.

Variables	Participants: *n* (%)	Exposure to pDDIs: *n* (%)			
		pDDIs of All Types	*p*-Value	Major pDDIs	*p*-Value
Gender ^a^					
Female	232 (54)	192 (44.7)	0.239	72 (16.7)	0.77
Male	198 (46)	172 (40)		64 (14.9)	
Age (years) ^a^					
18–60	197 (45.8)	171 (39.8)	0.255	53 (12.3)	0.05
>60	233 (54.2)	193 (44.9)		83 (19.3)	
Number of medications used per participant ^a^					
≤6	102 (23.7)	47 (10.9)	<0.001	6 (1.3)	<0.001
7–12	234 (54.4)	228 (53)		70 (16.3)	
≥13	94 (21.9)	89 (20.7)		60 (14)	
Hospital stays (days) ^a^					
≤3	235 (54.7)	172 (40)	<0.001	18 (4.2)	<0.001
≥4	195 (45.3)	192 (44.7)		118 (27.4)	
Number of comorbidities ^a^					
≤2	305 (70.9)	244 (56.7)	<0.001	66 (15.3)	<0.001
≥3	125 (29.1)	120 (27.9)		70 (16.3)	
Comorbidities/complications					
Hypertension ^a^	132 (30.7)	123 (28.6)	0.001	57 (13.3)	<0.001
Diabetes complications ^a^	71 (16.5)	61 (14.2)	0.74	23 (5.3)	0.87
CKD ^a^	48 (11.1)	41 (9.5)	0.87	19 (4.4)	0.20
CHF ^a^	39 (9.0)	33 (7.7)	0.99	18 (4.2)	0.04
CAD ^a^	39 (9.0)	35 (8.1)	0.35	14 (3.3)	0.54
AKI ^a^	35 (8.2)	34 (7.9)	0.03	12 (2.8)	0.72
Dyslipidemia ^a^	29 (6.7)	26 (6.0)	0.59	12 (2.8)	0.24
CSVD ^b^	29 (6.7)	27 (6.3)	0.28	16 (3.7)	0.005
Gastroenteritis ^b^	28 (6.5)	21 (4.9)	0.17	9 (2.1)	0.95
UTI ^b^	25 (5.8)	22 (5.1)	0.78	12 (2.8)	0.07

^a^ Pearson’s chi-square test. ^b^ Fisher’s exact test. Abbreviations: CKD = chronic kidney disease; CHF = congestive heart failure; CAD = coronary artery disease; AKI = acute kidney injury; CSVD = cerebrovascular disease; UTI = urinary tract infection. Diabetes complications include diabetic foot 32 (7.4%), diabetic neuropathy 28 (6.5%), diabetic nephropathy 6 (1.4%), and diabetic retinopathy 5 (1.1%).

**Table 2:** Logistic regression analysis based on exposure to all and major pDDIs.

Variables	All Types of pDDIs				Major-pDDIs			
	Univariate Analysis		Multivariate Analysis		Univariate Analysis		Multivariate Analysis	
	OR (95% CI)	*p*-Value	OR (95% CI)	*p*-Value	OR (95% CI)	*p*-Value	OR (95% CI)	*p*-Value
Gender								
Female	Reference				Reference			
Male	1.4 (0.8–2.4)	0.24	-	-	1 (0.7–1.6)	0.77	-	-
Age (years)								
18–60	Reference				Reference		Reference	
≥60	0.7 (0.4–1.2)	0.26	-	-	1.5 (0.9–2.2)	0.005	0.9 (0.5–1.5)	0.77
Drugs prescribed per participant								
≤6	Reference		Reference		Reference		Reference	
7–12	44.5 (18.1–109.3)	<0.001	30.1 (11.7–77.8)	<0.001	6.8 (2.9–16.3)	<0.001	2.1 (0.8–5.8)	0.12
≥13	20.8 (7.8–55.6)	<0.001	5.4 (1.6–19.1)	0.008	28.2 (11.2–71.2)	<0.001	5.5 (1.9–16.1)	0.002
Hospital stays (days)								
≤3	Reference		Reference		Reference		Reference	
≥4	23.4 (7.2–76)	<0.001	9.7 (2.5–38.7)	0.001	18.5 (10.6–32.3)	<0.001	11.3 (5.9–21.6)	<0.001
Number of comorbidities								
≤2	Reference		Reference		Reference		Reference	
≥3	6 (2.3–15.3)	<0.001	1.6 (0.5–5)	0.42	4.6 (2.9–7.2)	<0.001	1.4 (0.7–2.5)	0.25
Comorbidities/complications								
Hypertension	3.2 (1.5–6.7)	0.002	1.5 (0.5–3.7)	0.43	2.1 (1.3–3.2)	<0.001	1.2 (0.7–2.1)	0.39
Diabetes complications	1.1 (0.5–2.3)	0.74	-	-	1 (0.6–1.8)	0.88	-	-
CKD	1 (0.4–2.5)	0.87	-	-	1.5 (0.8–2.8)	0.21	-	-
CHF	0.9 (0.4–2.4)	0.99	-	-	1.9 (1–3.9)	0.04	2.1 (0.9–5.2)	0.08
CAD	1.6 (0.6–4.8)	0.36	-	-	1.2 (0.6–2.5)	0.54	-	-
AKI	6.7 (0.9–49.8)	0.06	5.4 (0.6–47.7)	0.13	1.1 (0.5–2.4)	0.72	-	-
Dyslipidemia	1.6 (0.5–5.5)	0.44	-	-	1.5 (0.7–3.4)	0.24	-	-
CSVD	2.6 (0.6–11)	0.20	-	-	2.9 (1.3–6.2)	0.007	1.6 (0.6–4.3)	0.30
Gastroenteritis	0.5 (0.2–1.3)	0.14	0.2 (0.6–0.9)	0.42	1 (0.4–2.3)	0.95	-	-
UTI	1.3 (0.4–4.6)	0.63	-	-	2 (0.9–4.7)	0.07	3.5 (1.1–10.6)	0.02

OR = odds ratio; CI = confidence interval; pDDIs = potential Drug–Drug Interaction.

**Table 3:** Clinical relevance, documentation and risk rating, and monitoring/management guidelines of top 10 major drug–drug interactions in participants with diabetes.

Interactions ^a^	Documentation and Risk Rating [31]	DIPS Score	Clinical Relevance		Monitoring and Management Guidelines [17,32]
			Signs and Symptoms ^a^	Abnormal Laboratory Results ^a^	
Ceftriaxone/ Calcium Containing products [30]	Fair D	5 (*n* = 4), 4 (*n* = 5), 3 (*n* = 22), 2 (*n* = 4)	Fever (10), Cough (7), Chest pain (1), Sepsis (1), Difficulties in breathing (6), Nephrolithiasis (1)	Leukocytosis (17), Evaluated BUN (16), High serum creatinine (13)	Ceftriaxone should not be administered concurrently with calcium-containing intravenous solutions, including continuous infusions such as parenteral nutrition administered via the Y-site. Avoid mixing them in the same IV administration line. Patients should be monitored for potential nephrotoxicity, thrombosis, precipitate deposition in the lungs, or reduced effectiveness of ceftriaxone.
Clopidogrel /Omeprazole [17]	Good X	4 (*n* = 1), 3 (*n* = 15), 2 (*n* = 1)	Shortness of breath (3), pedal edema (2), fatigue (1)	Evaluated D-dimer (1)	Avoid concurrent use of clopidogrel with omeprazole because that combination may result in decreased effectiveness of clopidogrel. Use of pantoprazole or rabeprazole as an alternative to omeprazole may reduce the risk.
Ramipril/ Spironolactone [14]	Good (C)	5 (*n* = 2), 4 (*n* = 2), 3 (*n* = 10)	Tachycardia (3), chest pain (2), vomiting (1), nausea (1), diarrhea (1)	Hyperkalemia (2), Hypokalemia (1) Hyponatremia (4), Evaluated BUN (5), High serum creatinine (6)	Patients should be closely monitored for persistent hyperkalemia, especially those with diabetes or renal dysfunction, as hyperkalemia may result in serious arrhythmia and be life-threatening. A spironolactone dose of 25 mg daily or on alternate days may be considered for patients co-prescribed with spironolactone and ramipril.
Spironolactone/ Candesartan [10]	Fair (C)	5 (*n* = 2), 4 (*n* = 1), 3 (*n* = 6), 2 (*n* = 1)	Tachycardia (2), chest pain (2), palpitation (1), vomiting (1), diarrhea (2), difficulties in breathing (1)	Evaluated T wave (2), Hyperkalemia (1), Hyponatremia (4), Evaluated BUN (1), High serum creatinine (1)	Patients should be closely monitored for persistent hyperkalemia, renal toxicity, and hypotension, especially those with diabetes and the elderly, because these conditions may result in serious arrhythmia and death. Dose adjustment or avoidance of concurrent use may be considered in high-risk patients to prevent serious complications
Clopidogrel/ Lansoprazole [9]	Fair C	3 (*n* = 8), 2 (*n* = 1)	Shortness of breath (1), pedal edema (1), chest pain (1)	Evaluated D-dimer (1)	Avoid concurrent use of clopidogrel and lansoprazole because that combination may decrease clopidogrel’s effectiveness. The use of pantoprazole or rabeprazole may decrease the risk alternative to lansoprazole.
Levofloxacin/ Sucralfate [8]	Excellent D	4 (*n* = 1), 3 (*n* = 6), 2 (*n* = 1)	Fever (1), urosepsis (1), sepsis (1)	Leukocytosis (5), neutrophilic leukocytosis (2).	Avoid concurrent use of levofloxacin and sucralfate, which reduces levofloxacin absorption, leading to decreased effectiveness. Sucralfate administration at least 2 h before or 2 h after taking levofloxacin. Monitor the patient for treatment response and ensure that the infection resolves as expected.
Aspirin/Ketorolac [6]	Fair X	5 (*n* = 1), 4 (*n* = 1), 3 (*n* = 4)	Tachycardia (1), basal pain with edema (1), blood seeping (1)	Thrombocytopenia (2), High PT (1)	Ketorolac can intensify the toxic effects of aspirin and elevate the risk of bleeding. Monitor patients’ platelet counts and assesses any signs of bleeding. If adverse effects are observed, reduce the dose of aspirin and consider using a proton pump inhibitor for gastrointestinal protection.
Insulin–aspart/ Pioglitazone [5]	Fair D	5 (*n* = 1), 3 (*n* = 4),	Tachycardia (1), nervousness or anxiety (1), fatigue (1)	Hypoglycemia (1)	Regular monitoring of blood glucose levels and clinical signs of hypoglycemia is essential, and insulin dose adjustments should be made as clinically indicated to maintain optimal glycemic control.
Spironolactone/ Ketorolac [5]	Fair C	5 (*n* = 1), 3 (*n* = 3), 2 (*n* = 1)	Severe headache (1), palpitations (1), chest pain (1), bradycardia (1)	Hypotension (1), Hyperkalemia (1), Hyponatremia (2), Evaluated BUN (2), High serum creatinine (1)	Ketorolac may reduce antihypertensive activity and enhance the hyperkalemic effect of Spironolactone. Patients should be monitored for hyperkalemia, worsening signs and symptoms of renal dysfunction, the efficacy of spironolactone, and blood pressure in those chronically using spironolactone with ketorolac and should be educated about the decrease in dietary potassium intake.
Allopurinol/ Ramipril [5]	Fair C	3 (*n* = 5),	Fever (2), bradycardia (2), acral edema (1)	Eosinopenia (1), Leukocytosis (2),	The concomitant use of allopurinol and ramipril increases the risk of hypersensitivity reactions. A patient receiving allopurinol along with ramipril should be closely monitored for signs of hypersensitivity following the start of allopurinol treatment for a period of no less than 5 weeks.

BUN = blood urea nitrogen; PT = prothrombin time. ^a^ Frequencies are given in parenthesis and calculated among participants with respective interactions.

## Data Availability

The data presented in this study are included in the article. Further inquiries can be directed to the corresponding author.

## References

[1] Aloke C., Egwu C.O., Aja P.M., Obasi N.A., Chukwu J., Akumadu B.O., Ogbu P.N., Achilonu I. (2022). Current advances in the management of diabetes mellitus. Biomedicines.

[2] Sun H., Saeedi P., Karuranga S., Pinkepank M., Ogurtsova K., Duncan B., Stein C., Basit A., Chan J., Atlas J.M.I.D. (2022). Global, regional and country-level diabetes prevalence estimates for 2021 and projections for 2045. Diabetes Res. Clin. Pract..

[3] Wahidin M., Achadi A., Besral B., Kosen S., Nadjib M., Nurwahyuni A., Ronoatmodjo S., Rahajeng E., Pane M., Kusuma D. (2024). Projection of diabetes morbidity and mortality till 2045 in Indonesia based on risk factors and NCD prevention and control programs. Sci. Rep..

[4] Demiral G.Z., Akın S.B. (2024). The effects of micro-and macrovascular diabetic complications on ischemic stroke in patients with type 2 diabetes mellitus. Turk. J. Neurol..

[5] Moke E., Demaki W., Daubry T., Ataikiru O., Agbonifo-Chijiokwu E., Ofulue O., Ekuerhare B., Akpoyovwere O., Edje K., Isibor N. (2023). Coexistence of hypertension with diabetes mellitus and its pharmacotherapy. Sci. Afr..

[6] Deol R., Bashir S. (2024). Exploring the complications of type 2 diabetes mellitus: Pathophysiology and management strategies. EPRA Int. J. Res. Dev. (IJRD).

[7] Xie X., Wu C., Hao Y., Wang T., Yang Y., Cai P., Zhang Y., Huang J., Deng K., Yan D. (2023). Benefits and risks of drug combination therapy for diabetes mellitus and its complications: A comprehensive review. Front. Endocrinol..

[8] Kulenovic A., Lagumdzija-Kulenovic A. (2023). Minimization of drug interactions in polypharmacy treatments of diabetes mellitus type 2 with cardiovascular comorbidities by using the decision support tool PM-TOM. Inform. Med. Unlocked.

[9] Min J.S., Bae S.K. (2017). Prediction of drug–drug interaction potential using physiologically based pharmacokinetic modeling. Arch. Pharmacal Res..

[10] Li L., Baker J., Quirk R., Deidun D., Moran M., Salem A.A., Aryal N., Van Dort B.A., Zheng W.Y., Hargreaves A. (2024). Drug–drug interactions and actual harm to hospitalized patients: A multicentre study examining the prevalence pre-and post-electronic medication system implementation. Drug Saf..

[11] Köhler G., Bode-Böger S., Busse R., Hoopmann M., Welte T., Böger R. (2000). Drug-drug interactions in medical patients: Effects of in-hospital treatment and relation to multiple drug use. Int. J. Clin. Pharmacol. Ther..

[12] Kovačević M., Vezmar Kovačević S., Miljković B., Radovanović S., Stevanović P. (2017). The prevalence and preventability of potentially relevant drug-drug interactions in patients admitted for cardiovascular diseases: A cross-sectional study. Int. J. Clin. Pract..

[13] Rasool M.F., Rehman A.u., Khan I., Latif M., Ahmad I., Shakeel S., Sadiq M., Hayat K., Shah S., Ashraf W. (2023). Assessment of risk factors associated with potential drug-drug interactions among patients suffering from chronic disorders. PLoS ONE.

[14] Syafhan N.F., Andrajati R., Wispriyono B., Noor S. (2024). Prevalence and Associated Factors of Drug-Drug Interactions in Hospitalized Diabetic Patients: A Cross-Sectional Study. J. Sains Farm. Klin..

[15] Makambwa E., Putra M.Y., Illahi A.D., Khan M.A., Yanuar A. (2025). Virtual screening of the Zimbabwe natural product database for glucokinase activators. Asian J. Pharm. Clin. Res..

[16] AL-Musawe L., Torre C., Guerreiro J.P., Rodrigues A.T., Raposo J.F., Mota-Filipe H., Martins A.P. (2020). Polypharmacy, potentially serious clinically relevant drug-drug interactions, and inappropriate medicines in elderly people with type 2 diabetes and their impact on quality of life. Pharmacol. Res. Perspect..

[17] Ren W., Liu Y., Jiang H., Lv X., Zhang N. (2024). Epidemiology of potential drug-drug interactions in hospitalized patients with type 2 diabetes mellitus in China: A retrospective study. Front. Endocrinol..

[18] Ahmad A. (2026). Assessing Knowledge Gaps and Awareness of Diabetes-Related Drug-Drug, Drug-Food, and Drug-Disease Interactions Among Pharmacy and Non-Pharmacy Students in Saudi Arabia. J. Young Pharm..

[19] Wiryani L.S.U., Putri E.M., Herman H., Prabowo P., Yuniarto P.F., Winartiana W. (2025). Analysis of Drug Use and Potential Interactions in Type 2 Diabetes Inpatients at a Type C Hospital, Kediri (2024). MEDFARM J. Farm. Dan Kesehat..

[20] Trevisan D.D., Silva J.B., Póvoa V.C., Araujo C.P., Oliveira H.C., Araújo E.P., Secoli S.R., Lima M.H.M. (2016). Prevalence and clinical significance of potential drug-drug interactions in diabetic patients attended in a tertiary care outpatient center, Brazil. Int. J. Diabetes Dev. Ctries..

[21] Ray C.-Y., Wu V.C.-C., Wang C.-L., Tu H.-T., Huang Y.-T., Kuo C.-F., Chang S.-H. (2021). Hypoglycemia associated with drug–drug interactions in patients with type 2 diabetes mellitus using Dipeptidylpeptidase-4 inhibitors. Front. Pharmacol..

[22] Tuladhar L.R., Shrestha S.L., Regmi D., Bimali S., Bhusal S., Khadka P. (2021). Drug-drug interactions between hypoglycemic and non-hypoglycemic medication in diabetic patients with comorbidities in a tertiary care center: A descriptive cross-sectional study. JNMA J. Nepal Med. Assoc..

[23] Dash R.P., Babu R.J., Srinivas N.R. (2018). Reappraisal and perspectives of clinical drug–drug interaction potential of α-glucosidase inhibitors such as acarbose, voglibose and miglitol in the treatment of type 2 diabetes mellitus. Xenobiotica.

[24] Ikäheimo I., Karjalainen M., Tiihonen M., Haanpää M., Kautiainen H., Saltevo J., Mäntyselkä P. (2019). Clinically relevant drug-drug interactions and the risk for drug adverse effects among home-dwelling older persons with and without type 2 diabetes. J. Clin. Pharm. Ther..

[25] Zanatta L., Dalla Cort F.N., Silva Mathias N., Argenta C. (2020). Analysis of drug interactions and epidemiological profile of individuals with diabetes mellitus in primary care. Rev. Enferm. UFSM.

[26] Whiteside H.L., Hillerson D., Buescher V., Kreft K., Mayer K.P., Montgomery-Yates A., Gupta V.A. (2022). Establishing a cardiac ICU recovery clinic: Characterizing a model for continuity of cardiac critical care. Crit. Pathw. Cardiol..

[27] Roblek T., Vaupotic T., Mrhar A., Lainscak M. (2015). Drug-drug interaction software in clinical practice: A systematic review. Eur. J. Clin. Pharmacol..

[28] Patel R.I., Beckett R.D. (2016). Evaluation of resources for analyzing drug interactions. J. Med. Libr. Assoc..

[29] Baxter C. (2010). Stockley’s Drug Interactions.

[30] Horn J.R., Hansten P.D., Chan L.-N. (2007). Proposal for a new tool to evaluate drug interaction cases. Ann. Pharmacother..

[31] Pandya N. (2007). Facts and comparisons 4.0. J. Med. Libr. Assoc..

[32] Bačar Bole C., Nagode K., Pišlar M., Mrhar A., Grabnar I., Vovk T. (2023). Potential drug-drug interactions among patients with schizophrenia spectrum disorders: Prevalence, association with risk factors, and replicate analysis in 2021. Medicina.

[33] Habib S.H., Saha S. (2010). Burden of non-communicable disease: Global overview. Diabetes Metab. Syndr. Clin. Res. Rev..

[34] Alemayehu T.T., Wassie Y.A., Bekalu A.F., Tegegne A.A., Ayenew W., Tadesse G., Getachew D., Yazie A.S., Teketelew B.B., Mekete M.D. (2024). Prevalence of potential drug–drug interactions and associated factors among elderly patients in Ethiopia: A systematic review and meta-analysis. Glob. Health Res. Policy.

[35] Upadhyay D. (2007). Pattern of potential drug-drug interactions in diabetic out-patients in a tertiary care teaching hospital in Nepal. Med. J. Malays..

[36] Santos-Díaz G., Pérez-Pico A.M., Suárez-Santisteban M.Á., García-Bernalt V., Mayordomo R., Dorado P. (2020). Prevalence of potential drug–drug interaction risk among chronic kidney disease patients in a Spanish hospital. Pharmaceutics.

[37] Aleksić D.Z., Milosavljević M.N., Stefanović S.M., Bukonjić A., Milosavljević J.Z., Janković S.M., Božović I., Perić S., Lavrnić D. (2021). Risk factors for potential drug–drug interactions in patients with myasthenia gravis. Neurol. Res..

[38] Castro-Granell V., Garin N., Jaén Á., Cenoz S., Galindo M.J., Fuster-RuizdeApodaca M.J. (2021). Prevalence, beliefs and impact of drug-drug interactions between antiretroviral therapy and illicit drugs among people living with HIV in Spain. PLoS ONE.

[39] Perić A., Udilović A., Dobrić S., Vezmar Kovačević S. (2022). The impact of treatment choices on potential drug–drug interactions in hypertensive patients. Br. J. Clin. Pharmacol..

[40] Navaratinaraja T.S., Kumanan T., Siraj S., Sreeharan N. (2023). Potential drug–drug interactions among hospitalised elderly patients in northern Sri Lanka, a lower middle-income country: A retrospective analysis. Drugs-Real World Outcomes.

[41] Ismail M., Khan S., Khan F., Noor S., Sajid H., Yar S., Rasheed I. (2020). Prevalence and significance of potential drug-drug interactions among cancer patients receiving chemotherapy. BMC Cancer.

[42] Humza A.U., Akbar M.A., Bilal Awan M.A., Yousuf J.B., Khan K., Ammar A. (2024). Prevalence and comparative analysis of potential drug-drug interactions among hospitalized patients at a tertiary care cardiac institute in Pakistan: Findings from a single centre. Pak. J. Pharm. Sci..

[43] Hamidah K.F., Rahmadi M., Meutia F., Kriswidyatomo P., Rahman F.S., Izzah Z., Zulkarnain B.S., Aminde L.N., Alderman C.P., Suprapti B. (2022). Prevalence and factors associated with potentially inappropriate medication and medication complexity for older adults in the emergency department of a secondary teaching hospital in Indonesia. Pharm. Pract..

[44] Zafar R., Rehman I.U., Shah Y., Ming L.C., Goh H.P., Goh K.W. (2023). Comparative analysis of potential drug-drug interactions in a public and private hospital among chronic kidney disease patients in Khyber Pakhtunkhwa: A retrospective cross-sectional study. PLoS ONE.

[45] Samadd M.A., Patwary F.T., Islam M.M., Munia A.T., Sikdar K.Y.K., Sarkar M.R. (2025). Risk Factors and Patterns of Drug-Drug Interactions in Two Categories of Level-3 Hospitals in Dhaka: A Cross-Sectional Study. Health Sci. Rep..

[46] Rabba A.K., Abu Hussein A.M., Abu Sbeih B.K., Nasser S.I. (2020). Assessing Drug-Drug Interaction Potential among Patients Admitted to Surgery Departments in Three Palestinian Hospitals. BioMed Res. Int..

[47] Noor S., Ismail M., Khadim F. (2020). Potential drug–drug interactions associated with adverse clinical outcomes and abnormal laboratory findings in patients with malaria. Malar. J..

[48] Milovanovic I.R., Pejcic A.V. (2025). Drug-drug interactions in hospitalized urological patients: A retrospective cohort study. Pharmacology.

[49] Haq I., Ismail M., Khan F., Khan Q., Ali Z., Noor S. (2020). Prevalence, predictors and outcomes of potential drug-drug interactions in left ventricular failure: Considerable factors for quality use of medicines. Braz. J. Pharm. Sci..

[50] Caratozzolo S., Gipponi S., Marengoni A., Pari E., Scalvini A., Pasina L., Magoni M., Padovani A. (2016). Potentially serious drug-drug interactions in older patients hospitalized for acute ischemic and hemorrhagic stroke. Eur. Neurol..

[51] Andrade C. (2021). The inconvenient truth about convenience and purposive samples. Indian J. Psychol. Med..

[52] Lobo M.G.A.d.A., Pinheiro S.M.B., Castro J.G.D., Momenté V.G., Pranchevicius M.-C.S. (2013). Adverse drug reaction monitoring: Support for pharmacovigilance at a tertiary care hospital in Northern Brazil. BMC Pharmacol. Toxicol..

[53] Patidar D., Rajput M.S., Nirmal N.P., Savitri W. (2013). Implementation and evaluation of adverse drug reaction monitoring system in a tertiary care teaching hospital in Mumbai, India. Interdiscip. Toxicol..

[54] Chowdhary R., Roshi T., Tandon V. (2022). Role of free web based software in evaluating the profile of drug-drug interactions. J. Cardiovasc. Dis..

